# Relationship between nomophobia and impulsivity among deaf and hard-of-hearing youth

**DOI:** 10.1038/s41598-022-17683-1

**Published:** 2022-08-20

**Authors:** Huda Shaaban Awed, Mohammad Ahmed Hammad

**Affiliations:** Special Education Department, Faculty of Education, Najran University, Najran, Saudi Arabia

**Keywords:** Psychology, Health care

## Abstract

Excessive use of smartphones is known to be associated with negative social, physical, and psychological outcomes across age groups. A related problem is called “no-mobile-phone phobia” or “nomophobia,” which is an extreme anxiety caused by not having access to a mobile phone. Despite their detrimental effects, smartphone use is highly prevalent among deaf/hard-of-hearing (DHH) individuals owing to their accessibility features. Therefore, it was deemed important to identify the prevalence of nomophobia in DHH youth and to examine the association between impulsivity and nomophobia. Gender-based differences in nomophobia and impulsivity were also examined. Data were collected from 187 DHH youth in Saudi Arabia using the Nomophobia Questionnaire and Barratt Impulsiveness Scale (short form). Findings revealed that 71.65% of the participants experienced severe nomophobia. While, nomophobia was more prevalent among female DHH youth than males, this difference was not observed for impulsivity. A linear regression analysis indicated that higher impulsivity was significantly associated with higher nomophobia in this sample. These findings suggest the importance of helping youth understand the disadvantages of smartphone use, and the consequences of their abuse or addiction to smartphones by incorporating this information into school curricula. Additionally, educating parents may help them monitor their children’s smartphone use more closely.

## Introduction

Communication and information technologies are hallmarks of our contemporary life, most notably, smartphones. According to Anshari et al*.*^1^, a smartphone is no longer just a mobile phone, it is an essential element in our lives. Its use has extended to different generations, especially children and youth, who have been referred to as the virtual generation of the world. Consequently, it is not only because of the entertainment it provides but also because of its applications that perform multiple tasks in standard time, ability to download numerous applications, as well as being a means of obtaining lots of news and information that has facilitated communication between individuals, overstepping time, and placing obstacles^2^. Furthermore, smartphones enable young individuals to perform multiple daily tasks such as connecting with others, texting, email verification, Internet browsing, shopping, games, and entertainment, all using a single device^3^. However, notwithstanding their potential utility, the excessive use and inability to control this use cause many problems^4^, including an excessive behavior pattern^5,6^. In this regard, Gonçalves et al*.*^7^, indicated that overusing a smartphone could be a risk factor to an individual’s health.

“Nomophobia” is a newly-emerged term related to excessive dependence on a phone. Short for no-mobile-phone phobia, it is characterized by the fear of losing one’s phone or not having access to a phone. Specifically, nomophobia is used to describe the anxiety, fear, and discomfort caused by loss of communication with others owing to smartphone loss or Internet crashes, especially for individuals accustomed to using these devices^8^. The concept first emerged in 2008, in a study on concerns experienced by mobile phone users, conducted by the UK Post Office on 2100 mobile phone users^3^. Since then, several researchers have attempted to determine its prevalence. However, owing to differences in methodology, sample, assessment tools, geographical location, etc., their findings vary greatly. Nevertheless, these studies suggest a moderate to high prevalence across contexts, with a reported prevalence rate ranging from 42 to 98%^3,9–12^. A recent meta-analysis^13^ of 20 research papers from ten countries reported a 70% prevalence rate for moderate to severe nomophobia. Despite varying rates, is evident that nomophobia is rapidly becoming a serious public health concern. Therefore, it is suggested that nomophobia should be included as an attitudinal phobia or as one of the types included under the definition of “social phobia” in the Fifth Edition of the Diagnostic and Statistical Manual of Mental Disorders (DSM-5)^14^.

According to Yildirim^3^, nomophobia involves several behaviors and symptoms that make an individual highly reliable and somniphobic, thereby making a mobile phone an extension of their bodies they cannot afford. Nomophobia has negative effects on both physical and psychological outcomes of young individuals^15^. According to Gezgin and Cakir^16^, in several cases, an individual begins to worry about forgetting their phone somewhere, running out of battery charge, losing network connectivity, disabling it, and being unable to use it. This constant state of anxiety leads a lack of concentration and problems such as dissatisfaction and loneliness when one is without one’s cell phone, frustration, despair, and loss of happiness, all accompanied by several physical manifestations, including dizziness, heartache, and stomach disorders^17,18^. Several researchers have compared nomophobia with other mental illnesses such as impulsive behavior disorder^14,19,20^, obsessive compulsive disorder^21^, panic disorder^8^, and anxiety and depression^22,23^, owing to the presence of social isolation, low self-control, lack of empathy, personality relationship disorders, low self-esteem, and neuroticism. Further, anxiety levels and frequency of neuro-personality traits have been found to increase with an increase in severity of the addiction^23^.

Among the personality characteristics and behavioral patterns that previous studies have examined in the context of excessive mobile phone use and/or nomophobia, impulsivity has been found to be closely linked to overuse of and a greater sense of reliance on mobile phones^18^. Researchers suggest that using a mobile phone may be one way to satisfy powerful motivations to relieve negative conscience in the short term, thus making it a strong predictor of sleep phobia^24^. Impulsivity entails an individual's inclination and willingness to respond to rapid, unplanned actions toward internal and external stimuli, without considering the negative consequences of their actions to the self or others^25^. Additionally, such individuals tend to seek instant gratification and are unable to control their emotions^26,27^. Thus, individuals with a high level of impulsivity could experience difficulties in postponing their mobile phone use, especially when experiencing negative emotions, because they tend to rely more on their mobile phones to alleviate such negative emotions in the short term. Accordingly, impulsivity may predict nomophobia^24^. In their study on Chinese university students, Mei et al*.*^18^ confirmed that high impulsivity was closely linked to excessive mobile phone use and reliance. Similarly, Smetaniuk^28^ purported that nomophobia, which results from mobile phone dependence, can be regarded as an impulse control disorder. In explaining the mechanism of how nomophobia develops, researchers suggest that individuals with a high level of impulsivity often prefer to use their mobile phone as an option for gratification without thinking about the consequences of the act^14,18,24,28^.

Considering the impact of excessive mobile phone use on young individuals’ mental health, it was deemed important to examine its prevalence among youth. Furthermore, it would be interesting to confirm if impulsivity is a predictor of nomophobia in this age group. However, among youth, the present study focused on those who are deaf/hard-of-hearing (DHH). The reason for limiting the target population to DHH youth was that the use of mobile phones is integral to their assimilation into the hearing community and to communication with other DHH and hearing individuals. DHH individuals use social media to integrate with the hearing community without feeling the stigma attached to their disability^29,30^. Smartphones have provided an alternative for youth who lack the confidence to communicate face-to-face^31^. Social networking using their phone also provides them with greater control, without having to reveal their true identities or disabilities. Specifically, smartphone applications, such as WhatsApp, WeChat, and Telegram, can help them overcome DHH-directed constraints^30^. However, excessive use also exposes them to nomophobia and smartphone addiction^32^. Considering the vital role that smartphones play in the lives of DHH individuals, and that they are consequently at risk of experiencing nomophobia, it was decided to further understand this issue among DHH youth and derive appropriate practice insights to protect them from the ill-effects of excessive smartphone use. Further, despite recent research interest in nomophobia among youth worldwide, this phenomenon has not yet been studied among DHH youth in the Arab world, especially Saudi Arabia. Therefore, the present study sought to address the following research questions:

1. What is the prevalence of nomophobia among DHH youth in Saudi Arabia?

2. Is impulsivity associated with nomophobia among DHH youth in Saudi Arabia?

## Methodology

### Participants

The sample for this study was recruited from the DHH youth population in Saudi Arabia. Specifically, 187 DHH youth were invited to participate, aged 17–20 years (mean age: M = 18.9 years, SD = 1.85 years). The DHH youth who were registered in integration programs in secondary schools were recruited from the cities of Najran, Asir, and Jazan in the south of the Kingdom of Saudi Arabia. A convenience sampling method was used in this study. The inclusion criteria were (1) being 16–20 years of age, (2) owning and actively using a smartphone, and (3) being able to follow verbal or sign language instructions. Most of the DHH participants had severe to profound hearing loss (> 81 dB HL), and their hearing level was verified based on formal school reports. None of the participants had any other disabilities. They communicated through sign language and spoken language. No information regarding other forms of disability or cochlear implants was obtained from the selected DHH youth.

### Measures

#### Nomophobia Questionnaire (NMP-Q)

NMP-Q is a 20-item scale developed by Yildirim and Correia^33^. It comprises four factors (a) Not being able to communicate, (e.g., “I would feel nervous because I would not be able to receive text messages and calls”), (b) Losing connectedness, (e.g., “I would feel weird because I would not know what to do”), (c) Not being able to access information, (e.g., “Being unable to get the news on my smartphone would make me nervous”), and (d) Giving up convenience, (e.g., “If I could not use my smartphone, I would be afraid of getting stranded somewhere”). All 20 items are rated on a seven-point Likert-type scale ranging from 1 (strongly disagree) to 7 (strongly agree). The total score ranges from 20 to 140. A higher score on the NMP-Q reflects a higher level of nomophobia, with severity of nomophobia classified as follows: 0–20: no nomophobia, 21–59: mild, 60–99: moderate, and ≥ 100: severe nomophobia^33^. The NMP-Q has been shown to have high internal consistency (α = 0.95) and construct validity (r = 0.710)^3,33^. Previous studies applying the NMP-Q have confirmed its internal consistency (with a Cronbach’s alpha above 0.60), and extrinsic validity in samples from various countries like Portugal^15^, Italy^34^, China^17^, Pakistan^35^, Spain^7^, and Iran^36^. In the present study, the Cronbach’s α for all the four subscales of the Arabic version ranged from 0.80 to 0.89 in the sample of DHH youth, indicating excellent reliability.

#### Barratt Impulsiveness Scale short form (BIS-15)

The 15-item short form of the Barratt Impulsiveness Scale (BIS-15)^37^ is an abbreviated version of the 30-item Barratt Impulsiveness Scale (BIS-11)^38^. The BIS-15 is a self-reported questionnaire that assesses the frequency of impulsive thoughts and behaviors on a scale of one (rarely/never) to four (almost always), and it is often used in non-clinical populations^38,39^. The range of responses lead to total points with three points gained from analysis of factors. The subscale on attentional impulsiveness includes five statements and represents the speed of making decisions (e.g., “I am restless at lectures or talks.”). Motor impulsiveness comprises five statements and focuses on actions based on motivations without thinking of outcomes (e.g., “I act on the spur of the moment.”). The non-planning impulsiveness subscale includes five statements representing non-planning works for the future (e.g., “I plan tasks carefully.”). Total scale scores range from 15 to 60 points, with higher scores indicating the presence of impulsive behavior. The validity of BIS-15 has been confirmed based on a positive association with other self-report impulsivity measures and with behavioral impulsivity tasks^40–45^. In the present study, the Cronbach’s α for the Arabic version ranged from α = 0.79 to 0.87 for DHH youth, indicating excellent reliability.

### Scale translation procedures

For use in the present study, the NMP-Q and BIS-15 were translated from English to Arabic and then back-translated to English by language experts to ensure that the Arabic translation maintained the same meaning as the original questionnaire. First, the English version was translated into Arabic by an Arabic–English bilingual professor from the Department of English at the researchers’ institution. The Arabic version was then back-translated into English by another professor who specializes in English and whose first language is Arabic. Subsequently, the translated Arabic and English versions of the scale were reviewed by three specialists majoring in Arabic, Psychology, and English, respectively. Based on consensus among the three specialists, some words and items were revised to create the final Arabic version of the two tools.

As the target population for the present study comprised DHH youth, the questionnaire instructions and items were also translated into sign language, which was often their preferred mode of communication. Three teachers who worked with DHH youth were employed to administer the questionnaires. They were fluent in sign language and had had several daily interactions with the present participants. The researchers conducted a brief 40-min training to explain certain terms, such as nomophobia and impulsivity, to these sign-language teachers to enable them to communicate their meaning effectively to the DHH youth. Subsequently, the teachers were asked to peruse the questionnaires, and the researchers and teachers agreed on how to translate them into sign language. As DHH individuals are known to experience difficulties with verbal language, prior to the main data collection, a pilot study was conducted with seven DHH youth with different levels of language ability. They were asked to point out difficult words and items and suggest easier alternatives. These opinions were later incorporated by the researchers and specialists who translated the scale from English to Arabic and sign language.

### Data collection procedure

Data were collected in September 2021. Prior to data collection, all participants completed informed consent forms that highlighted that their participation was voluntary, that all information obtained would be kept strictly confidential, and that the data would only be used for research purposes. Before its commencement, the study received ethical approval from the Deanship of Scientific Research at Najran University (NU/-/SEHRC/10/1086) and the Special Education Department of the Education Department. Additionally, all procedures were carried out complying to the Declaration of Helsinki. Each participant provided informed consent. The questionnaires were administered to all the DHH youth in their schools. The data collection procedure took 60 min, after which participants received small gifts as a token of appreciation.

### Data analysis

All statistical analyses were conducted using SPSS version 20. Frequency and percentage were used to examine the prevalence of nomophobia among the DHH youth. In addition, means, standard deviations, and t-tests were used for bivariate analyses considering demographic variables. Finally, linear regression analysis was used to examine the association between impulsivity and nomophobia.

## Results

### Nomophobia and impulsivity

Table 1 shows the prevalence of nomophobia among the present sample of DHH youth. A large majority of the participants (71.65%) exhibited severe nomophobia, followed by about a quarter of the sample (23.52%) who exhibited moderate nomophobia. Rest of the participants (4.81%) exhibited mild nomophobia.

**Table 1:** Prevalence of nomophobia among DHH youth.

NMP-Q scores	Nomophobia level	Frequency (N = 187)	Percentage (%)
20	No nomophobia	0	0.0
> 20 to < 60	Mild nomophobia	9	4.81
60 to < 100	Moderate nomophobia	44	23.52
≥ 100	Severe nomophobia	134	71.65

*NMP-Q* Nomophobia Questionnaire.

### Association between nomophobia and impulsivity

The association between nomophobia and impulsivity was examined using linear regression. Prior to the regression analysis, several bivariate analyses were conducted to identify demographic variables that could influence this association. Findings revealed significant differences only in the nomophobia levels across genders. Table 2 presents the findings of the bivariate analysis examining gender differences on NMP-Q and BIS-15 scores. As the two gender groups were unequal and the Levene’s test for equality of variances was not met, Welch’s t-test was used to examine this variable. Findings revealed statistically significant differences (p < 0.001) between males and females on the NMP-Q total score and on its subscales, with t-values ranging from 2.59 to 3.97, and moderate effect sizes for all variables (Cohen’s d 0.4–0.6). Specifically, across all subscales of the NMP-Q and on the total score, female DHH youth exhibited a higher mean score as compared to males. There were no significant differences between males and females in terms of impulsivity.

**Table 2:** Means (M), standard deviations (SD), and t values for nomophobia and impulsivity among DHH youth.

Variables	Male (N = 118)	Female (N = 70)	t	P	Effect size
**Nomophobia**					
Not being able to communicate	22.62 (5.82)	25.43 (6.01)	3.16	0.002**	0.5
Losing connectedness	26.21(5.68)	28.43 (5.44)	3.63	0.01*	0.4
Not being able to access information	33.90 (7.41)	36.51 (5.35)	2.59	0.01*	0.4
Giving up convenience	26.59 (5.80)	29.34 (4.38)	3.45	0.001**	0.5
Total	109.14 (19.66)	119.72 (13.94)	3.97	0.00**	0.6
**Impulsivity**					
Attentional impulsiveness	14.46 (3.95)	13.97 (3.20)	0.89	0.37^NS^	0.1
Motor impulsiveness	14.43 (3.13)	14.44 (3.18)	0.20	0.98^NS^	0.0
Non-planning impulsiveness	14.04 (3.47)	13.93 (3.14)	0.22	0.82^NS^	0.0
Total	42.35 (6.43)	42.34 (7.42)	0.009	0.99^NS^	0.0

Significant at *p < 0.05, **p < 0.01.

*NS* not significant.

No significant results were observed for any other demographic variable. Therefore, it was decided to include only gender as a control variable in the linear regression analysis. As evident from Table 3, after controlling for the effect of gender differences, impulsivity explained 11% of the variance in nomophobia severity in the present sample of DHH youth. Rest of the variance can be attributed to other factors, which need to be examined in future studies.

**Table 3:** Linear regression analysis results of association between impulsivity and nomophobia.

Variables	B	Std. error	Beta	t	P
Constant	67.91	7.77		8.73	0.00
Impulsivity	0.962	0.194	0.22	4.94	0.00

R = 0.34; R^2^ = 0.117; adjusted R^2^ = 0.112; F (24.48) p < 0.05. R = 0.22, R^2^ = 0.049, F (9.55) p < 0.05.

## Discussion

High mobile phone usage among DHH youth is understandable because smartphones have several advantages, such as high-resolution photography, easy recording of videos and lessons, easy downloading of substantial amounts of data from books without having to carry a textbook, connecting with others without geographical and time restrictions, and easy interaction on social media. Moreover, owing to the migration to virtual education during the current circumstances of the Coronavirus Disease 2019 (COVID-19) pandemic DHH youth could communicate with their teachers and friends online, thus allowing them to pursue their lessons without interruption. These factors may contribute to DHH youth’ dependence on mobile phones as an integral part of their lives, leading to behavioral addiction to the device.

The present findings revealed a high prevalence of severe and moderate nomophobia among DHH youth in Saudi Arabia (71% and 24%, respectively). This was evident from a range of behaviors assessed by the NMP-Q, including attachment to the device and behavioral addiction to it, anxiety about Internet outages and being outside the range of communication reception, repeatedly checking the device to avoid missing a text message or call, disconnection from family and friends owing to mobile phone use, and nervousness resulting from battery consumption. Indeed, the negative effects of excessive mobile phone use, as suggested by previous studies, was also evident in the present sample. Based on NMP-Q scores, participants’ fear of not having access to a mobile phone was evident, which interfered with their social relationships and face-to-face communication. In terms of the prevalence rates observed, interestingly, despite being limited to DHH youth, the present sample exhibited rates that were comparable to those reported in previous studies. For instance, a recent meta-analysis^13^ of 20 research papers from ten countries reported a 70% prevalence rate for moderate to severe nomophobia, while another study reported a 98% prevalence in Turkey^9^.

The present results also indicated differences in nomophobia levels between males and females, which females exhibiting a higher prevalence of nomophobia. This finding is consistent with several studies that indicated a higher incidence of nomophobia among females^3,16,31^. However, it is also in contrast with other studies that reported higher incidence of nomophobia among males^11,46^. Therefore, it is important to consider the contextual and socio-cultural factors when interpreting the current findings. In Saudi Arabia, males are considered to have more freedom to socialize as compared to females. Therefore, the latter tend to prefer to use their smartphones for social media activities, entertainment, and shopping; thus, leading to their excessive use of mobile phones^47^.

Further, Pavithra et al*.*^48^ reported that females tended to create more than one account on different social media applications such as Facebook, Twitter, Instagram, Snapchat. This was found to be associated with the tendency to check their phones frequently to remain updated about new notifications, and thus to the resultant increase their excessive attachment to mobile phones^48^. The gender difference in nomophobia incidence could also be explained by the different entertainment/leisure preferences of males and females. A study on Turkish university students reported that males tend to seek entertainment through sports or electronic sports, while females prefer engaging in social and cultural activities^49^. It could be purported that, due to the multiplicity of alternatives in pursuing sports, such as playing sports at the club, electronic games through the computer, or recreational activities with friends, males may be less attached to mobile phones as compared to females. Conversely, smartphones are an efficient way to connect with others, owing to the wide range of social networking apps available. Therefore, it may be likely that females gravitate more towards mobile phone use for their preferred leisure activities.

The present results also indicated that there was no gender-based difference in impulsivity among this sample of DHH youth, which corroborates the findings of similar studies on non-DHH samples^50,51^. The present result may be explained by the fact that the causes of impulsivity may be common across DHH individuals. Specifically, when one examines the causes of hearing loss and their relationship to brain damage, educational disability and communication imposed by hearing loss, and the resulting frustration, high impulsivity is expected^52^. Further, common personality characteristics among DHH youth, including inability to concentrate, to review and resist pressures resulting from a problem, poor planning and executive function, desire for instant gratification, emotion avoidance, intolerance to criticism, and anxiety are all indicative of impultivity^53^. Similarly, poor self-regulation, overactivity, lack of calmness, lack of thought about results before taking actions, and lack of self-control have been found to co-exist with impulsivity^54^. These characteristics are commonly observed among DHH individuals^55–57^.

Finally, the present results suggested an association between nomophobia and impulsivity, such that the latter explained 11% of the variance in nomophobia after controlling for gender difference. This can be explained by the fact that nomophobia is characterized by the tendency to engage in an activity, such as smartphone overuse, without thinking about its consequences^18^, a characteristic that is often observed in individuals with high impulsivity^58^. Further, high mobile phone use has been found to be a strong predictor of impulsivity^59^ and nomophobia^59,60^. Considering that mobile phone use has increased exponentially in the past two years owing to the COVID-19 pandemic and the related social isolation and online learning, DHH individuals have also been spending long periods using a mobile phone. Based on this, one can surmise that the increase in mobile phone use may have resulted in high impulsivity and nomophobia in the present sample.

## Conclusions and implications

The main objective of this study was to identify the prevalence of nomophobia in DHH youth and to examine the association between impulsivity and nomophobia. The findings revealed that moderate to severe nomophobia was prevalent among DHH youth, with significant gender-based differences. Specifically, females exhibited higher levels of nomophobia as compared to males. In addition, results from the linear regression analysis indicated that impulsivity predicted 11% of the variance in nomophobia, after controlling for gender differences. This also highlights the fact that other factors influence the level of nomophobia among DHH youth in Saudi Arabia, which needs to be examined further in future studies. Specifically, the role of the family, peers, community, and socio-cultural factors needs to be explicated.

Extant research provides substantial evidence for the negative impact of mobile phone overuse and nomophobia on individuals physical and psychological well-being. Therefore, it is important to acknowledge this as a public health concern and to devise appropriate strategies to control mobile phone dependence and nomophobia across all age groups. Specifically, youth is a sensitive stage for the development of socio-emotional skills and future mental health outcomes. DHH youth face additional challenges such as pressure to assimilate with the hearing population, and face stigma and stereotyping owing to their disability. Therefore, the negative consequences of mobile phone dependence and nomophobia could have a severe effect on their short- and long-term well-being.

The present results suggest the importance of helping youth understand the disadvantages of smartphone use, and the consequences of their abuse or addiction to smartphones. This could be done by incorporating this information into school curricula, for instance, within health education programs. Additionally, educating parents may help them monitor their children’s smartphone use more closely, thus combining family interventions with more formal treatment programs. In addition, psychological counseling programs that combine cognitive behavioral techniques, as well as client- and family-centered practices could help mitigate the ill-effects of mobile phone dependence. Finally, smartphone technologies themselves could be used to control smartphone overuse through programs such as those which alert the user about prolonged use and help limit screen time.

## Limitations and future directions

Despite revealing findings that provide important practice insights for professionals and family members of DHH youth, the present study had several limitations. Firstly, this was a cross-sectional study, which limited inferences on causality. Future longitudinal and qualitative studies will shed more light on the variables studied. Secondly, the study did not include a control or comparison group. Including a matched sample of hearing youth could have revealed interesting findings on the factors influencing nomophobia among both groups. Further, due to resource limitations, the present sample had to be recruited using convenience sampling. However, considering the feasibility of the study and the specificity of the target population (DHH youth), it was difficult to utilize a more robust quantitative sampling method. Another limitation caused by the resource crunch was dependence on single informants. Using multiple stakeholder perspectives, such as those of parents and teachers of DHH youth, could have yielded deeper explanation of the mechanism of development of nomophobia and influencing factors. Regarding the scale used to assess nomophobia, the authors would like to acknowledge that a validated Arabic version of the scale has been developed by Al-Balhan et al.^61^. However, this validated translation could not be obtained for the present study. Therefore, the authors proceeded with translating the tool again. Though rigorous methods were used to translate and back-translate the tool from English to Arabic, and to verify the appropriateness of the translation, it is important to note that the lack of tool validation may have influenced the present results. This point needs to be considered when interpreting and using the current findings. Finally, with reference to factors associated with nomophobia, in the present sample, impulsivity only explained 11% of the variance in nomophobia. The rest of the 89% of the variance could be attributed to several other factors, including but not limited to role of the family, peers, community, and socio-cultural factors specific to DHH youth. Future studies need to deconstruct the individual and combined effect of these factors on both impulsivity and nomophobia among DHH youth.

## Data Availability

The dataset generated during and/or analyzed during the current study is available from the corresponding author on reasonable request.
